# The Seat of Life in Man

**Published:** 1898-12

**Authors:** 


### Selection.

### THE SEAT OF LIFE IN MAN.

THE recent visit of Dr. Rudolph Virchow, the German biologist, to England, and his lecture before the Charing Cross Hospital Medical School, were events of no little interest. This eminent investigator is now seventy-seven years old, and yet age has scarcely touched him. Except for a somewhat feeble voice, one would hardly detect any evidence of weakness in his manner or appearance. On the occasion of his lecture he was honored by the presence on the platform of Lord Lister, Sir Henry Thompson, Sir Douglas Galton, Sir William Huggins, Sir John Evans and a number of other scientific men, nearly all of whom were septuagenarians. Rarely are so many persons distinguished for their services to civilisation seen together in England, outside of the meetings of the Royal Society and the British Association for the Advancement of Science.

Professor Virchow spoke in the language most familiar to his audience, and his enunciation was excellent. It was remarked at the time that it was doubtful whether any Englishman of the same age could address a French or German audience in a similar manner with as much finish and success.

The greatest achievement of Virchow is probably his study and reduction to order of the varied morbid growths called "tumors" and his classification of them with reference to the age of the patient. But he has investigated many other phases of disease, and has also taken an active part in municipal and national politics. He was an alderman in Berlin, and has occupied a seat in the Prussian Landtag.

### CELLULAR PATHOLOGY.

Professor Virchow's name has been closely associated in the scientific world with a theory of disease which makes the cells the seat of life, and consequently of all disorders. He is sometimes spoken of, indeed, as the father of "cellular pathology"—as this doctrine is called. The old notion was that man is a unit in his existence; but the modern theory is that each cell has its own life, and that man is, therefore, an association of living beings. In fact, he is believed to bear substantially the same relation to the cells as a nation does to its citizens. He is a grand composite, not an individual. To deal with disease, then, one must aim to affect the cells of the organ which is involved. Professor Virchow's lecture in London early in the month was largely a reassertion of this doctrine.

Paracelsus, the medieval philosopher, conceived that life was the work of a special "spiritus," and acted upon matter from without as upon a machine. Francis Glisson, as Professor Virchow remarked the other day, was one of the first scientific men to teach that life was inherent in matter. Unfortunately, said the lecturer, Glisson failed to distinguish between animate and inanimate matter. He regarded "irritability" or the disposition to respond in some ways when excited by touch or otherwise, the chief characteristic of matter.

Another old school of scientists held that the seat of life was the blood, and that all disease was caused by trouble with that fluid. This theory, known as "humoral pathology," was discarded by Huxley and Virchow at about the same time. Huxley was then studying life in the Charing Cross Hospital, where fifty years later the now venerable German biologist offers tribute to his memory.

Professor Virchow added: "The greatest difficulty in the advance of biology has been the natural tendency of its disciples to set the search after the unity of life in the forefront of their inquiries. Hence arose the doctrine of vital force, an assumption now discarded, but still revealing its influence from time to time in isolated errors. No satisfactory progress can be made till the idea of highly organised living things as units has been set aside; till it is recognised that they are in reality organisms, each constituent part of which has its special life. Ultimate analysis of higher animals and plants brings us alike to the cell, and it is these single parts, the cells, which are to be regarded as the factors of existence. The discovery of the development of complete beings from the ova of animals and the germ cells of plants has bridged the gap between isolated living cells and complete organisms, and has enabled the study of the former to be employed in elucidating the life of the latter. In a medical school, where the teaching is almost exclusively concerned with human beings, this sentence should be writ large: 'The organism is not an individual, but a social mechanism.' Two corollaries must also be stated—(1) that every living organism, like every organ and tissue, contains cells; (2) that the cells are composed of organic chemical substances, which are not themselves alive."

### PROFESSOR SCHWANN'S MISTAKE.

In amplifying the cell theory, the lecturer remarked that Schwann, a famous German physiologist, who died in 1882, erred in supposing that certain growths of a diseased character, if not of healthy tissue, could proceed freely without any parental cells. This belief ignored the distinction between living and nonliving matter, and amounted to "spontaneous generation." But by long observation and experiment Professor Virchow was able to extend to disease a maxim that was already well established otherwise, that all life must come from antecedent life. Or, to put the idea in modern language, every cell owes its origin to some other cell.

It is admitted that a large class of diseases are the product of microbes, or parasites, that invade the system. But apart from infection, Professor Virchow attributes disease to the inherited properties of the cells of the organ affected. However, he is careful to avoid pushing the notion of inherited disease too far. He calls attention, for instance, to the once widely prevalent belief that leprosy was transmitted from parent to child by heredity. But as soon as the specific microbe of leprosy was discovered, that idea was abandoned. It became evident that infection, not heredity, explained the later cases.

In regard to infection, the lecturer remarked that parasites act upon their victims in two ways. The larger ones (by which, perhaps, he meant cancers,) devour parts of their hosts, while the smaller ones secrete a virulent poison. The recognition of this latter fact has led to the brilliant work of Lister on the one hand, and to the introduction of serum therapeutics on the other.

The speaker referred at length to the benefits conferred on mankind by antiseptic surgery and closed as follows:

It remains for me to say a word concerning the other great problem, the solution of which the whole world is awaiting with anxious impatience. I refer to the problem of immunity and its practical corollary, artificial immunisation. It has already happened once that an Englishman has succeeded in applying this to the definite destruction of at least one of the most deadly infectious diseases. Jenner's noble discovery has stood its trial as successfully, except in popular fancy, as he hoped. Vaccine is in all hands; vaccination is, with the aid of governments, spreading continually.

Pasteur also labored with determination; others have followed him, and the new doctrine of antitoxins is continually acquiring more adherents. But it has not yet emerged from the conflict of opinions, and still less is the secret of immunity itself revealed.—*New York Tribune.*

---

### ARGONIN IN MIDDLE-EAR CATARRH.

Gray (*Texas Courier-Record of Med.*) says that he has found argonin more serviceable than boric acid in acute suppuration of the middle ear, especially where there are small perforations in the membrane, attended with high inflammation. He gives his experience in detail and concludes as follows: The advantages of argonin over boric acid in acute middle ear catarrhs may be thus briefly summarised:

1. Argonin solution is highly antiseptic; boric acid, if at all, very slightly so.

2. Argonin in solution can be forced through a small perforation in the drumhead, thus reaching every part of the tympanic cavity and Eustachian tube. In such a case boric acid lies inactive in the external auditory canal.

3. Argonin can be used to flush the middle ear and tube, thus reaching every part of the inflamed tract, carrying out with it all product of inflammation.

4. Argonin excites a decided and positive effect upon the suppurative processes. Boric acid possesses this power but feebly.

5. Argonin stimulates the closing of perforations in the drumhead. Boric acid has no such action.

